# Predicting Risk of Motor Vehicle Collisions in Patients with Glaucoma: A Longitudinal Study

**DOI:** 10.1371/journal.pone.0138288

**Published:** 2015-10-01

**Authors:** Carolina P. B. Gracitelli, Andrew J. Tatham, Erwin R. Boer, Ricardo Y. Abe, Alberto Diniz-Filho, Peter N. Rosen, Felipe A. Medeiros

**Affiliations:** 1 Visual Performance Laboratory, Department of Ophthalmology, University of California, San Diego, California, United States of America; 2 Department of Ophthalmology, Federal University of São Paulo, São Paulo, São Paulo, Brazil; 3 Princess Alexandra Eye Pavilion and Department of Ophthalmology, University of Edinburgh, Edinburgh, United Kingdom; 4 Entropy Control, Inc., La Jolla, California, United States of America; 5 Department of Ophthalmology, University of Campinas, Campinas, São Paulo, Brazil; 6 Department of Ophthalmology and Otorhinolaryngology, Federal University of Minas Gerais, Belo Horizonte, Minas Gerais, Brazil; Universidade Federal do Rio de Janeiro, BRAZIL

## Abstract

**Purpose:**

To evaluate the ability of longitudinal Useful Field of View (UFOV) and simulated driving measurements to predict future occurrence of motor vehicle collision (MVC) in drivers with glaucoma.

**Design:**

Prospective observational cohort study.

**Participants:**

117 drivers with glaucoma followed for an average of 2.1 ± 0.5 years.

**Methods:**

All subjects had standard automated perimetry (SAP), UFOV, driving simulator, and cognitive assessment obtained at baseline and every 6 months during follow-up. The driving simulator evaluated reaction times to high and low contrast peripheral divided attention stimuli presented while negotiating a winding country road, with central driving task performance assessed as “curve coherence”. Drivers with MVC during follow-up were identified from Department of Motor Vehicle records.

**Main Outcome Measures:**

Survival models were used to evaluate the ability of driving simulator and UFOV to predict MVC over time, adjusting for potential confounding factors.

**Results:**

Mean age at baseline was 64.5 ± 12.6 years. 11 of 117 (9.4%) drivers had a MVC during follow-up. In the multivariable models, low contrast reaction time was significantly predictive of MVC, with a hazard ratio (HR) of 2.19 per 1 SD slower reaction time (95% CI, 1.30 to 3.69; P = 0.003). UFOV divided attention was also significantly predictive of MVC with a HR of 1.98 per 1 SD worse (95% CI, 1.10 to 3.57; P = 0.022). Global SAP visual field indices in the better or worse eye were not predictive of MVC. The longitudinal model including driving simulator performance was a better predictor of MVC compared to UFOV (R^2^ = 0.41 *vs* R^2^ = 0.18).

**Conclusions:**

Longitudinal divided attention metrics on the UFOV test and during simulated driving were significantly predictive of risk of MVC in glaucoma patients. These findings may help improve the understanding of factors associated with driving impairment related to glaucoma.

## Introduction

Driving is the primary source of transportation in many countries and is therefore considered an instrumental activity of daily living for many people. [1] Previous studies have shown that ability to drive is intimately related to health-related quality of life, [2–5] with driving cessation associated with increased risk of depression, social isolation and entry into residential care. [2,4] On the other hand, driving is a highly visual task, and continued driving in the presence of significant visual impairment is likely to be associated with increased risk of motor vehicle collisions (MVC), with potentially serious implications for the individual and society. [6–11]

Glaucoma is a leading cause of visual impairment in the world. [12] Previous studies have shown that drivers with glaucoma are at increased risk of MVC. [13–17] Haymes and colleagues reported drivers with glaucoma to be over 6 times more likely to have been involved in a MVC compared to similarly aged controls. [13] However, previous studies have shown only a weak association between MVC and visual field defects as assessed by standard automated perimetry (SAP). [13–19] In SAP, the ability to detect a static white-on-white peripheral visual stimulus at threshold is evaluated under optimal conditions of adaptation and testing. However, these artificial test conditions minimize potential distractions and may give unrealistic estimates of the amount of useful vision that is available to a complex task such as driving.

In contrast to the traditional visual field test, an assessment of visual performance under conditions of divided attention would better reflect the demands imposed by driving, when subjects are frequently required to detect or monitor a peripheral stimulus, while simultaneously attending to a central task. [20–23] The Useful Field of View (UFOV) test (Visual Awareness, Inc, Chicago, IL) is a computer-based assessment developed to evaluate processing speed under conditions of divided attention and previous studies have suggested this test to be a useful predictor of risk of MVC. [21,22,24–26] In addition, the ability to divide attention may also be tested under simulated scenarios using driving simulators. By more closely reproducing the demands of real world driving, driving simulators may potentially allow better estimation of risk. [27]

In glaucoma patients, the relationship between MVC and measurements of divided attention from UFOV and driving simulation has only been evaluated using retrospective data. [19,25] Therefore, although studies have reported impaired ability to divide attention to be associated with increased odds of a history of MVC, their retrospective design did not allow for prediction of individual risk. Furthermore, patients may behave differently on UFOV or driving simulation after occurrence of a MVC due to modified driving behaviours, leading to difficulties in establishing a causal relationship. A further limitation of previous studies is that UFOV and simulation data were acquired only at a single point in time. It is possible that longitudinal changes on these tests could provide even stronger predictive ability compared to single point observations. To our knowledge, such prospective assessment of the relationship between longitudinal changes on divided attention tests and risk of MVC has not been yet evaluated in patients with glaucoma.

The purpose of the current study was to determine whether longitudinal UFOV and driving simulator measures were predictive of risk of MVC in a cohort of glaucomatous subjects followed over time. In addition, we compared the predictive ability of these tests to those of standard functional assessments conducted in this population.

## Methods

This was a longitudinal observational cohort study. Subjects were recruited as part of the Diagnostic Innovations in Glaucoma Study: Functional Impairment, conducted at the Visual Performance Laboratory, Department of Ophthalmology, University of California San Diego (UCSD). Written informed consent was obtained from all participants, and the institutional review board and human subjects committee at the UCSD prospectively approved all methods. All study methods adhered to the tenets of the Declaration of Helsinki for research involving human subjects and the study was conducted in accordance with the regulations of the Health Insurance Portability and Accountability Act.

At each visit during follow-up, patients underwent a comprehensive ophthalmologic examination, review of medical history, contrast sensitivity assessment using the Pelli-Robson contrast sensitivity chart (Precision Vision, La Salle, IL), stereoscopic optic disc photograph (Kowa Nonmyd WX3D, Kowa Optimed, Inc, Torrance, CA), and SAP using the Swedish interactive threshold algorithm (SITA Standard 24–2, Carl Zeiss Meditec, Inc., Dublin, CA, USA). Only patients with open angles on gonioscopy were included. Subjects were excluded if they had any other ocular or systemic disease that could affect the optic nerve or the visual field. Subjects also completed a Montreal Cognitive Assessment (MOCA), which is a test developed to detect mild cognitive impairment, similar to the Mini-Mental State Examination. [28]

Glaucoma was defined by the presence of repeatable (≥ 3 consecutive) abnormal SAP tests. Eyes with documented evidence of progressive glaucomatous optic disc changes noted on masked grading of stereophotographs were also classified as glaucomatous, regardless of visual field findings. SAP tests were defined as abnormal if they had a pattern standard deviation with P <0.05 and/or a glaucoma hemifield test result outside normal limits. Visual fields with more than 33% fixation losses or false-negative errors, or more than 15% false-positive errors, were excluded. In order to evaluate binocular visual field loss, sensitivities of the monocular SAP tests of the right and left eyes were combined to calculate an integrated binocular visual field. The sensitivity for each point of the binocular visual field was estimated using the binocular summation model described by Nelson-Quigg et al. [29]

### Useful Field of View (UFOV)

The UFOV was used to assess visual processing speed (in milliseconds [ms]) under divided attention. Details of the test have been provided previously. [24–26] In brief, participants were asked to identify a silhouette of a 2 cm by 1.5 cm truck or car (2 choices), that appeared in a box on the center of a 17-inch touchscreen monitor, in addition to a concurrent peripheral localization task (an image of a car presented on one of eight radial spokes at a fixed eccentricity of approximately 11 degrees). The subject was then asked to identify the central target and to identify on which spoke the outside object was located. The test proceeded with decreasing presentation times, ranging from 500 ms to 17 ms, until the presentation time that would result in a 75% accurate response could be recorded. All stimulus presentations were at maximum contrast for UFOV divided attention task. All subjects had prior experience with the UFOV test, having performed at least one test previously.

### Driving Simulation

The ability to divide attention was also assessed by measuring reaction times to stimuli presented during a divided attention protocol during simulated driving. Details of the methodology employed for driving simulation have been provided previously.^7,^ [30] The test evaluated the ability to attend simultaneously to a central visual task of driving (negotiating curves on a winding country road for 4 minutes) and to a peripheral visual task of perceiving a projected stimulus and responding by pushing a button on the steering wheel. The peripheral stimuli were presented at about 20-degrees of visual angle in the upper right and upper left of the simulator screen. The contrast of the stimulus was altered using alpha blending techniques to achieve symbol transparencies of 0.1 and 0.9. Therefore in the case of 0.1 symbol transparency, the symbol intensity and color that the driver perceived was 10% of the symbol intensity and color and 90% of the background intensity and color. The equivalent Michelson contrasts were 0.04 and 0.27 for low- and high-contrast stimuli, respectively. At maximum screen intensity the divided attention stimulus symbols were pure white, while the background was constant and consisted of a cloudy sky. Examples of the size and shape of targets has been presented in detail elsewhere. [19,30] For each contrast level, there were an average of 5 stimuli presented which stayed on the screen for a maximum of 3 to 6 seconds (uniform distribution) or until the driver responded. The next stimuli appeared between 3 and 6 seconds (again uniform distribution) after the driver responded or when the maximum display time had elapsed.

The main outcome measure of reaction time was defined as the time interval between appearance of the peripheral stimulus and the subject pressing the button on the steering wheel, with a longer reaction time indicating worse result. Reaction times have been extensively used in the psychophysical literature for investigation of visual processing speed. [27,31] The mean reaction time was calculated by averaging the reaction time for all presentations at specific contrast levels (low and high). We denominated these metrics as “low contrast reaction time” and “high contrast reaction time”. A false positive percentage was calculated, defined as the number of button presses occurring when no stimulus had been presented divided by the total number of stimuli presented. [30]

The overall reaction time to a visual stimulus may also be influenced by the motor response (the act of pressing a button). However, it is expected that the motor response contribution to the reaction time will be similar to different contrast presentations. The difference in reaction times to detect two stimuli under different contrasts should depend essentially on the ability of the visual system to detect the stimuli, while the motor response to press the button should stay essentially the same once the stimulus is detected. Therefore, we subtracted the overall reaction times from the low and high contrast stimuli in order to isolate the visual processing speed contribution to the overall reaction time. We denoted this metric as “corrected reaction time”.

As a patient might achieve fast reaction times to peripheral stimuli by adopting a strategy in which the central driving task is neglected, it was important to assess central driving task performance. [32] This was measured using “curve coherence”, which was defined as the normalized cross-correlation function between the road curvature (k_road_) and the vehicle path curvature (k_own_) as a function of spatial shift. Curve coherence was measured using the following equation:

$$curvecoherence=\underset{\text{arg}\left(delay\right)}{\text{max}}\left\{\frac{1}{n}\sum_{t}\frac{\left(k_{own}\left(t\right)-mean_{k_{own}}\right)\left(k_{road}\left(t,delay\right)-mean_{k_{road}}\right)}{SD_{k_{own}}SD_{k_{road}}}\right\}$$

Where *n* was the number of samples of the two signals and *SD* was the standard deviation of the signals, with a coherence of 1 indicating the two signals to be an exact match. The maximum coherence is computed across all delay shifts. To minimize the effect of unreliable tests and learning effect, all subjects underwent training prior to test commencement. Training consisted of a 2-minute practice of acceleration and deceleration, followed by 1 minute of the curve negotiation task.

### Follow-up and Definition of Study Endpoints

Subjects had UFOV and driving simulator tests performed at baseline and at every 6 months during follow-up. Prospective information (i.e., after baseline) regarding incidence of MVC during follow-up was obtained from the California Department of Motor Vehicles (DMV). The study endpoint was defined as the date of MVC reported on the DMV records. To evaluate whether longitudinal measurements were predictive of the study endpoints, only tests acquired before the MVC event date were analysed in the study. Subjects who did not experience a MVC were considered censored at the last follow-up visit. All tests up to the last available follow-up date were analysed for these patients. In short, only performance assessment data collected before an MVC was used in the analyses reported herein.

### Statistical Analysis

The primary purpose of this study was to determine whether longitudinal driving simulator metrics and UFOV divided attention tests were predictive of MVC in drivers with glaucoma. Hazard ratios (HRs) for the association between potential predictive factors and MVC were obtained by a Weibull survival model incorporating time-dependent covariates. [33] We report univariable as well as multivariable models adjusting for age, degree of cognitive impairment (MOCA score), severity of binocular visual field defect and average mileage driven per week.

As the magnitude of a hazard ratio for a particular variable depends on its unit of measurement, a direct comparison of hazard ratios would be an inappropriate way of comparing the ability of models in predicting risk of MVC. For this purpose, we used the modified R^2^ index proposed by Royston, [34] which has previously been used for similar purposes. [35,36] The modified R^2^ index is equivalent to the coefficient of determination of a linear model and measures the amount of variation in the outcome (survival time) explained by the predictors, or, in other words, the strength of the relationship between the predictors and the outcome in a survival model. The modified R^2^ index has been proposed as the best way to assess prognostic information of survival models. [37]

All statistical analyses were performed with commercially available software (Stata, version 13; StataCorp LP, College Station, TX.). The α level (type I error) was set at 0.05.

## Results

**Fig 1:**
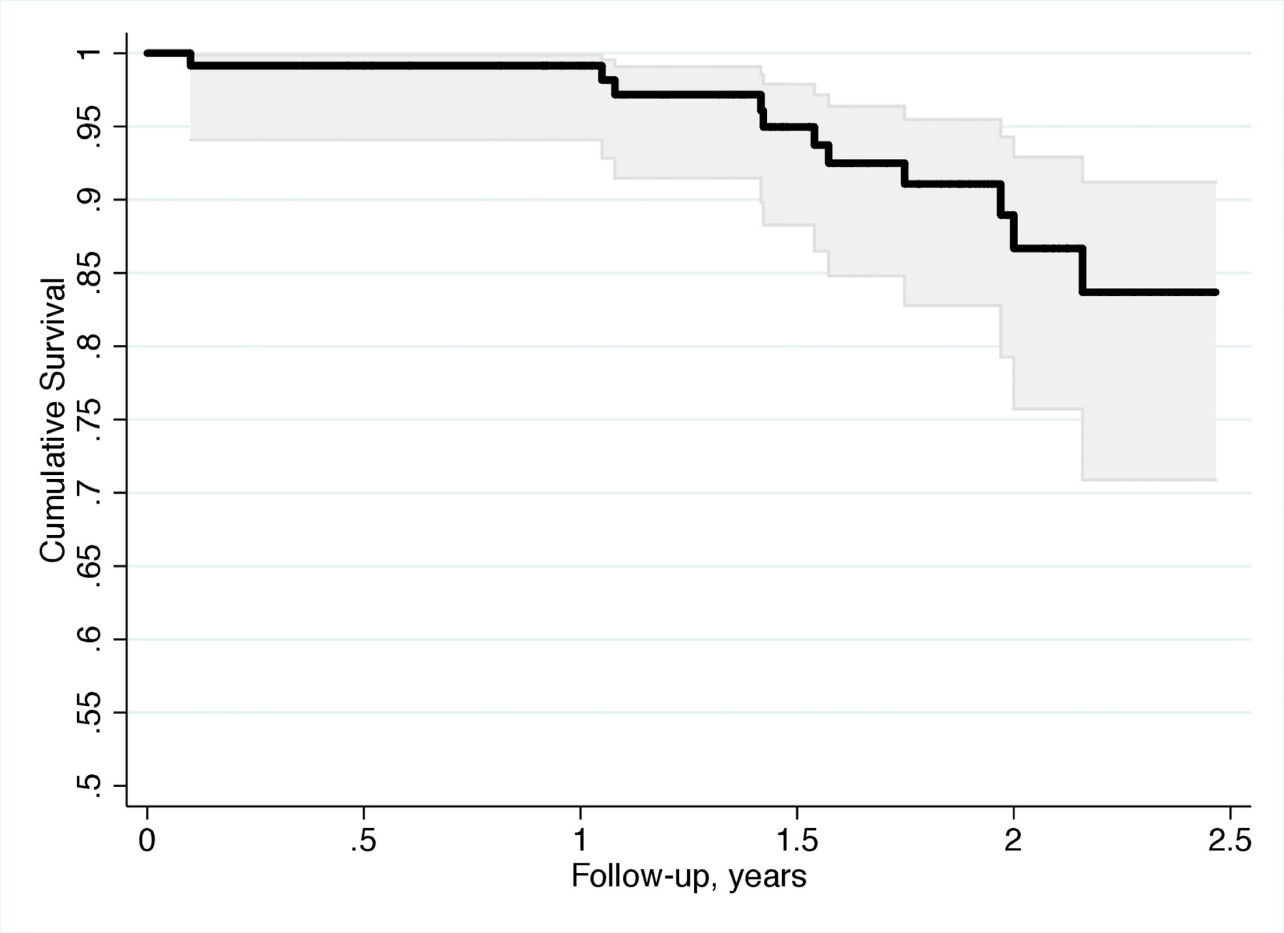
Kaplan-Meier survival curve estimating the cumulative probability of motor vehicle collision during follow-up.

The study included 117 subjects with glaucoma with a mean ± standard deviation age of 64.5 ± 12.6 years at baseline. Subjects were followed for an average of 2.1 ± 0.5 years with an average of 3.1 ± 2.3 visits during follow-up. 11 patients (9.4%) had at least one MVC during follow-up. Fig 1 illustrates the cumulative probability of having a MVC during the study. Table 1 shows baseline demographic and clinical characteristics of subjects who had MVC versus those who did not. At baseline, only the parameter measuring performance on the central driving task (curve coherence) was significantly different between the two groups. The individual characteristics of patient are shown in S1 Table.

**Table 1:** Baseline demographic and clinical characteristics of study patients (mean ± standard deviation).

Variables	No Motor Vehicle Collisions (106 subjects)	Motor Vehicle Collisions (11 subjects)	P-value
**Age (years)**	65.1 ± 12.0	58.5 ± 17.3	0.251
**Sex, female (%)**	42 (39.6%)	7 (63.6%)	0.198*
**Ethnicity (%)**			0.556*
**Caucasian**	67 (63.2%)	8 (72.7%)	
**African-American**	26 (24.5%)	1 (9.1%)	
**Other**	13 (12.3%)	2 (18.2%)	
**MD Worse Eye (dB)**	-3.4 ± 4.9	-3.5 ± 4.8	0.892
**MD Better Eye (dB)**	-1.1 ± 2.8	-2.0 ± 3.8	0.365
**Binocular SAP Sensitivity (dB)**	29.0 ± 2.3	28.7 ± 3.0	0.933
**Visual Acuity Worse Eye (LogMAR)**	0.06 ± 0.15	0.12 ± 0.23	0.850
**Visual Acuity Better Eye (LogMAR)**	-0.03 ± 0.11	-0.05 ± 0.11	0.588
**Contrast Sensitivity Worse Eye**	1.43 ± 0.21	1.40 ± 0.19	0.566
**Contrast Sensitivity Better Eye**	1.52 ± 0.15	1.49 ± 0.14	0.526
**Average Distance Driven per Week (miles)**	167 ± 224	178 ± 87	0.171
**Montreal Cognitive Assessment Score**	28 ± 3	28 ± 3	0.563
**UFOV Divided Attention Test (ms)**	71 ± 98	94 ± 149	0.785
**Low Contrast Reaction Time (s)**	0.94 ± 0.76	1.09 ± 1.37	0.929
**High Contrast Reaction Time (s)**	0.59 ± 0.19	0.58 ± 0.14	0.515
**Corrected Reaction Time (s)**	0.35 ± 0.70	0.51 ± 1.36	0.725
**Curve Coherence**	0.96 ± 0.01	0.91 ± 0.03	0.009

Abbreviations: MD = mean deviation, dB = decibels; logMAR = logarithm of the minimum angle of resolution; UFOV = Useful field of view; ms = miliseconds, s = seconds.

*Fisher’s exact test.

Table 2 reports hazard ratios (HRs) for univariable models with 95% confidence intervals (CI) for each putative predictive factor. In the univariable model, low contrast reaction time was significantly associated with higher risk of MVC during follow-up. Each 1 standard deviation (SD) slower low contrast reaction time was associated with a 57% increased risk of MVC (HR = 1.57 per 1 SD slower; 95% CI, 1.07 to 2.29; P = 0.020). Slower corrected reaction time was also associated with increased risk of MVC (HR = 1.57 per 1 SD larger; 95% CI, 1.08 to 2.30; P = 0.019), as well as worse performance on the central driving task (curve coherence) (HR = 1.50 per 1 SD lower 95% CI, 1.17 to 1.92; P = 0.001). Additionally, slower performance on the UFOV divided attention test (HR = 1.64 per 1SD slower; 95% CI, 1.01 to 2.65; P = 0.046) was associated with an increased risk of MVC during follow-up. Binocular visual field mean sensitivity, better eye MD, and worse eye MD were not associated with increased risk of MVC (Table 2).

**Table 2:** Results of univariable models for prediction of motor vehicle collisions in glaucoma patients.

Variables	Hazard Ratio	95% CI	P-value
Low contrast reaction time (per 1 SD slower)	1.57	1.07–2.29	0.020
High contrast reaction time (per 1 SD slower)	1.06	0.55–2.04	0.869
Corrected reaction time (per 1 SD larger)	1.57	1.08–2.30	0.019
Curve coherence (per 1 SD lower)	1.50	1.17–1.92	0.001
UFOV divided attention (per 1 SD slower)	1.64	1.01–2.65	0.046
MD worse eye (per 1 SD lower)	0.97	0.56–1.68	0.903
MD better eye (per 1 SD lower)	0.81	0.51–1.28	0.364
Binocular SAP sensitivity (per 1 SD lower)	0.93	0.52–1.69	0.820
Age (per 1 SD older)	0.64	0.40–1.04	0.069
Sex (Female)	2.90	0.85–9.93	0.089
Ethnicity (Caucasian)	1.43	0.38–5.38	0.600
Montreal Cognitive Assessment Score (per 1 SD lower)	1.11	0.59–2.11	0.742
Visual acuity worse eye (per 1 SD worse)	1.28	0.78–2.12	0.335
Visual acuity better eye (per 1 SD worse)	0.86	0.52–1.44	0.565
Contrast sensitivity worse eye (per 1 SD worse)	0.83	0.39–1.77	0.621
Contrast sensitivity better eye (per 1 SD worse)	0.87	0.50–1.52	0.621
Average mileage per week (per 1 SD further)	1.08	0.60–1.95	0.795

Abbreviations: SD = standard deviation.

**Fig 2:**
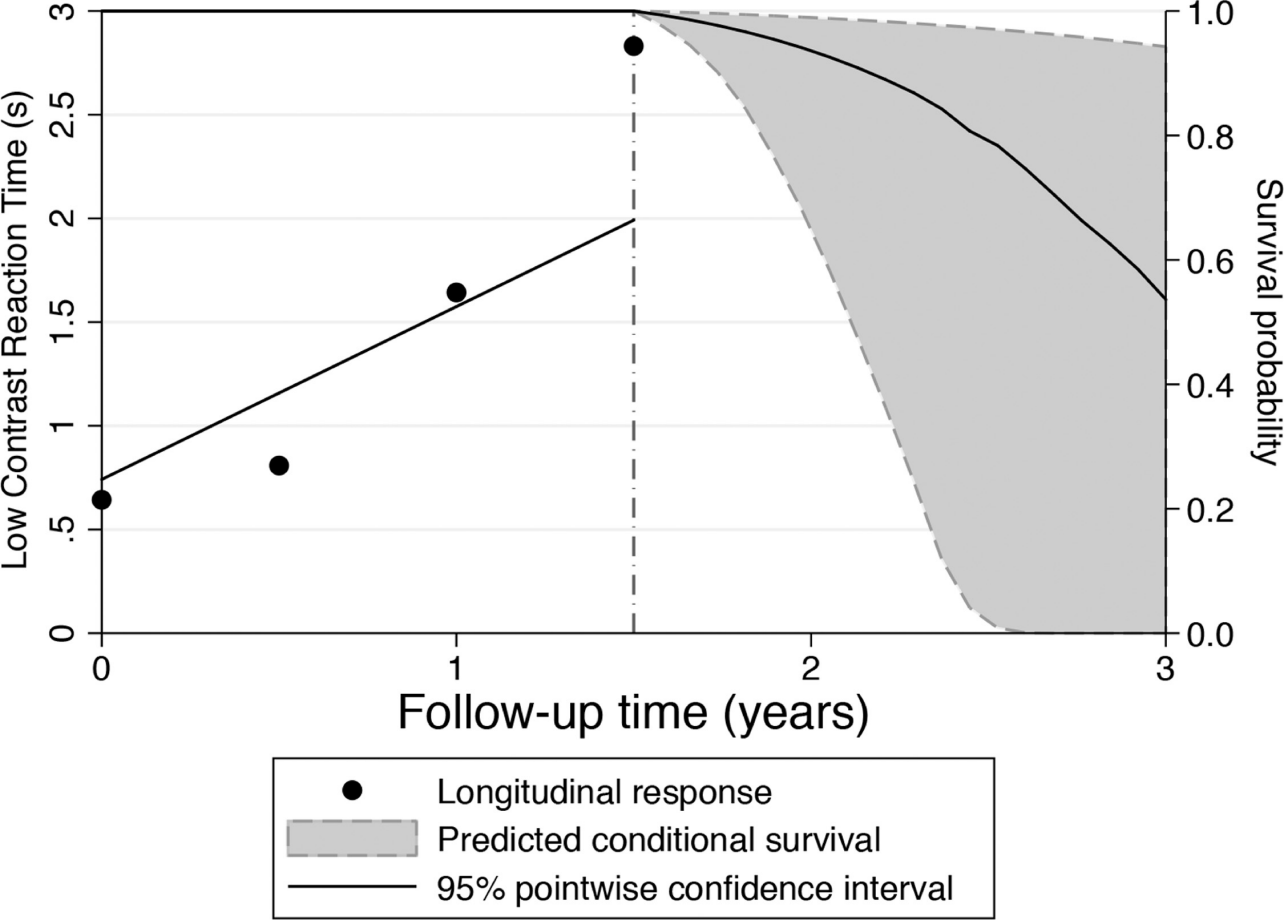
Predicted survival probabilities for a glaucoma patient that had a motor vehicle collision during follow-up. This patient demonstrated progressively slower reaction times during follow-up and relatively low predicted survival probabilities, indicating high risk of motor vehicle collision.

**Fig 3:**
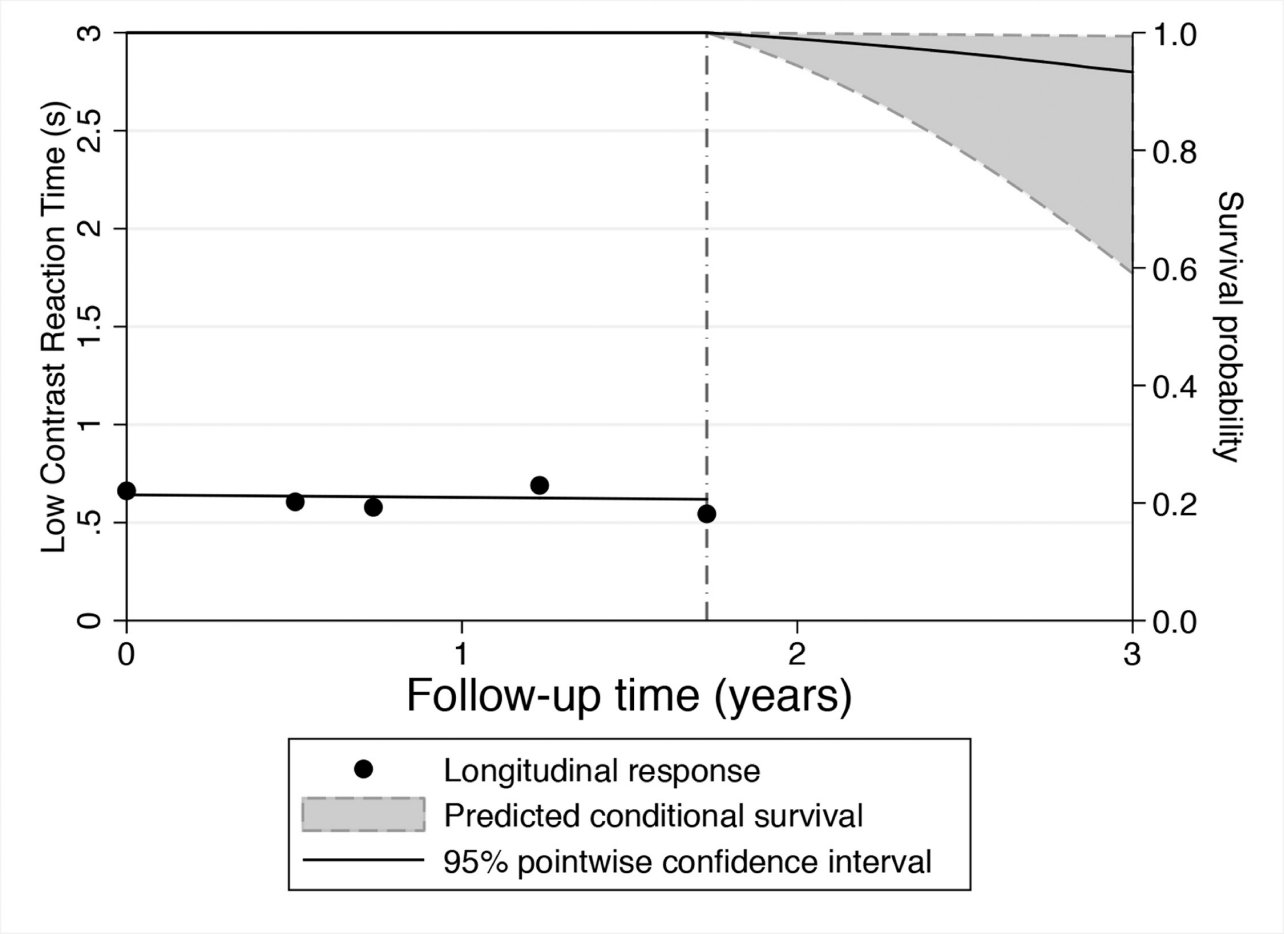
Predicted survival probabilities for a glaucoma patient that did not have a motor vehicle collision during follow-up. This patient demonstrated stable and relatively fast reaction times during follow-up, which resulted in high predicted survival probabilities and low risk of motor vehicle collision.

Table 3 shows multivariable HRs after adjusting for age, cognitive impairment (MOCA score), binocular SAP sensitivity, and average mileage driven per week. Driving simulator metrics of low contrast reaction time and curve coherence remained significantly associated with increased risk of MVC. Each 1 SD slower low contrast reaction time was associated with a 2.19 times increased risk of MVC (HR = 2.19 per 1 SD slower; 95% CI, 1.30 to 3.69; P = 0.003), whereas each 1 SD lower curve coherence increased the risk by 1.36 times (HR = 1.36 per 1 SD lower; 95% CI, 1.02 to 1.83; P = 0.039). The multivariable model including low contrast reaction time and curve coherence had an R^2^ of 41% for predicting risk of MVC. Figs 2 and 3 shows cases of glaucoma patients with different outcomes predicted by the longitudinal evaluation of driving simulator low contrast reaction times. Fig 2 shows a patient who demonstrated progressively slower reaction times during follow-up. Predicted survival probabilities for this patient were relatively low, indicating a relatively high risk of MVC. This subject in fact had a DMV-reported MVC during follow-up. Fig 3 shows a patient with relatively faster and stable reaction times, which resulted in high survival probabilities and low risk of MVC. This subject did not show evidence of MVC during follow-up.

**Table 3:** Results of multivariable survival analyses examining the relationship between driving simulation and UFOV metrics with risk of motor vehicle collisions, after adjustment for confounding factors.

DRIVING SIMULATION				USEFUL FIELD OF VIEW			
	HR	95% CI	P-value		HR	95% CI	P-value
**Low Contrast Reaction Time (per 1 SD slower)**	2.19	1.30–3.69	0.003	**UFOV Divided Attention (per 1 SD slower)**	1.98	1.10–3.57	0.022
**Curve Coherence (per 1 SD lower)**	1.36	1.02–1.83	0.039				
**MOCA Score (per 1 SD lower)**	1.40	0.66–2.96	0.386	**MOCA Score (per 1 SD lower)**	1.18	0.62–2.23	0.619
**Binocular SAP sensitivity (per 1 SD lower)**	0.88	0.44–1.76	0.714	**Binocular SAP sensitivity (per 1 SD lower)**	0.85	0.44–1.63	0.628
**Age (per 1 SD older)**	0.57	0.31–1.03	0.065	**Age (per 1 SD older)**	0.54	0.31–0.92	0.023
**Average Mileage per Week (per 1 SD further)**	1.30	0.71–2.38	0.399	**Average Mileage per Week (per 1 SD further)**	1.06	0.56–2.02	0.862

Abbreviations: SD = standard deviation; HR = Hazard Ratio.

Slower UFOV divided attention time also remained associated with increased risk of MVC in the multivariable model. Each 1 SD slower performance on the UFOV divided attention test was associated with 1.98 times increased risk of MVC (HR = 1.98 per SD slower, 95% CI, 1.10 to 3.57; P = 0.022). The multivariable model including UFOV had a R^2^ of 18% in predicting risk of MVC.

We also enrolled a group of 50 healthy subjects with normal optic disc evaluation and normal SAP results. None of the controls had a motor vehicle collision during a follow-up period of 2.1 ± 0.5 years. At the last follow-up visit, patients with glaucoma had significantly slower average low contrast reaction time on the simulator task compared to healthy subjects (1.22 ± 1.07s vs 0.61 ± 0.20s, respectively; P <0.001). In addition they also had significantly slower performance on the UFOV divided attention test (103 ± 129ms vs. 47 ± 57ms, respectively; P = 0.006).

## Discussion

This study has shown longitudinal performances on divided attention metrics to be independently predictive of risk of MVC in drivers with glaucoma. Both UFOV and driving simulator measures were predictive of MVC; however, after adjusting for confounding factors, driving simulator tasks had superior predictive ability compared to UFOV. To our knowledge this is the first longitudinal study to prospectively evaluate risk of MVC in drivers with glaucoma and the results provide evidence for the use of divided attention metrics as potential tools to assess risk of driving impairment in this population.

At baseline, UFOV divided attention and driving simulator low contrast reaction times were similar between MVC and no MVC groups, however drivers that went on to experience a MVC had worse performance on the central driving task of following a curve in the road (curve coherence). Worse central driving task performance was sustained during follow-up, but a significant difference in driving simulator low contrast reaction times also emerged between drivers with and without a MVC. Therefore, survival models incorporating longitudinal divided attention metric data helped to predict future occurrence of MVC in a way that was not possible using baseline metrics alone. Drivers who developed MVC also had larger corrected reaction times on driving simulation, which indicates that the difference in performance between groups was not likely to be due to differences in the motor response component of the driving simulator reaction times. [30]

Previous studies have indicated that drivers with glaucoma are at increased risk of MVC compared to those without the disease. [13],[30], [38], [39] Haymes et al [13] reported that the results of UFOV divided attention test were the strongest factor associated with MVC in glaucomatous patients (adjusted odds ratio = 10.29; 95% CI, 1.10–96.62). However, these studies relied on retrospective collection of MVC data and so were not able to assess the ability of different tests to predict risk of future MVC for individual patients. By prospectively collecting longitudinal data from predictive tests and incidence of MVC, the present study has addressed this limitation. The use of survival models allowed us to quantify the ability of the different tests to predict MVC, while also taking into account the censored aspect of the data and adjusting for the effect of confounding factors. Each 1 SD slower low contrast reaction time was associated with a 2.19 times higher risk of MVC in a multivariable model adjusting for age, curve coherence, MOCA score, binocular SAP and average distance driven per week. Furthermore, these models allowed individual prediction of risk, as illustrated in Figs 2 and 3. A subject with slower reaction times, as shown on Fig 2, was predicted to have a relatively higher risk (lower survival probability) of MVC, compared to a subject with faster reaction times, as shown on Fig 3. The use of longitudinal information allows updating the risk profile as patients are followed over time, resulting in improved estimates of risk.

Although the follow-up for the study was on average only 2.1 years, it is important to note that when assessing a driver’s risk of MVC, it is in fact the relatively short-term risk that is of most concern. In other words, it is more important to know if a driver has an increased risk of an MVC occurring in the near term, such as in one to three years, so that appropriate measures can be taken to mitigate the risk. The results of this study suggest that if one were to detect worsening ability to perform the UFOV or driving simulator tasks, indicating increased risk of MVC; one might be able to intervene to reduce risk. However, further studies are necessary to determine the level of risk of MVC deemed acceptable for continued driving, and the appropriateness of interventions to decrease risk, for example, restriction or revocation of driving license. It is also important to examine the influence of drivers’ self-perceived risk of MVC, and determine whether modifying driving behaviours, such as limiting driving in inclement weather or at night might reduce risk in higher risk individuals.

In agreement with previous cross-sectional studies we found conventional measures of visual function, including visual acuity and binocular SAP sensitivity had relatively low ability to predict MVC. [25,27] A possible explanation is that tests such as SAP are performed under artificial conditions and do not reflect the visual complexity of real-world driving. SAP is performed under conditions of minimal visual distraction, whereas the ability to deal with visual distractions, or to divide attention, is essential for most daily activities, including complex cognitively demanding activities such as driving. It should be noted, however, that our study included a relatively small proportion of patients with moderate or severe visual field damage. In addition, it is possible that specific types of visual field defects might be associated with increased risk of MVC. [40,41] For example, the study performed by Glen et al. showed that simulated superior visual field defects had more impact than inferior defects on MVCs. [41] The purpose of the current study was to validate driving simulator metrics that could be predictive of risk of real-life MVCs. Once these metrics are validated and can serve as surrogates for risk of MVC, one can then investigate the impact of different conditions on these metrics, such as how different patterns of visual field loss affect performance on the simulator metrics, and this should be the subject of future studies.

A likely reason for the better ability of the driving simulator to predict MVCs was that it better replicates the complexity of real driving situations, especially with inclusion of a divided attention task. The driving simulator also allowed testing using a more demanding low contrast divided attention stimuli, and the reaction times to low contrast stimuli were stronger predictors of MVC than reaction times to high contrast stimuli. This supports the findings of Tatham et al that drivers with glaucoma are affected more by low contrast tasks than similarly aged controls. [30]

Our study had limitations. As MVCs are relatively rare events, there were only a relatively small number of patients in the study who experienced a MVC during follow-up. The incidence of MVCs in the included glaucomatous population was higher than in a group of healthy subjects followed over time, a finding in agreement with previous studies. [42] Incidences of MVCs in different studies may vary according to the characteristics of the population studied, geographic location and methods of measurement, among other factors. However, to our knowledge, our study was the first to prospectively evaluate risk of MVC in a well-defined cohort of patients with glaucoma and we found statistically significant and clinically relevant results for divided attention variables, despite the relatively small number of MVCs. Due to the relative small numbers of MVCs in our cohort, it was also not possible to assess whether specific types or circumstances of MVCs were more common in patients with glaucoma and their relationship with the metrics investigated. It is possible that some of the MVCs were underreported to the DMV, especially minor incidents. According to California law, a driver must report a collision only if the damage caused is greater than $750, and /or anyone was injured or killed. However, use of DMV records is a useful method to reduce the potential for bias or underreporting that may occur when such information is obtained by self-report. With regard to the tests used to predict MVC evaluated in our study, it is important to note that driving simulators have limitations, as although they provide realistic, standardized scenarios, drivers may behave differently in real-world driving conditions. In addition, simulators may not be readily available for testing in clinical practice. Despite this, our findings may serve to validate driving simulation as a method for assessing driving impairment and also increase the understanding of the functional deficits that increase risk of MVC in patients with glaucoma. It is important to note that we excluded patients with motion sickness who were unable to complete driving simulation from the study. Motion sickness during driving simulation can potentially confound data, influence participant dropout rates and also limit the effectiveness of the test [43] [44] However, it is unlikely that this would have biased the results as there does not seem to be a relationship between simulator sickness and risk of MVC.

Another limitation of our study is the potential for confounding effects caused by media opacities, such as cataract. It is possible that cataract could result in worse performance on the tests evaluated in the study and also in higher incidence of MVC. However, the presence of cataract would likely be also detected by measurements of visual acuity, Pelli-Robson contrast sensitivity and SAP MD. The lack of significant effect of these variables suggests that cataract did not play a significant role as a confounder in our results.

In conclusion, this study found that longitudinal driving simulator metrics and UFOV divided attention test were independent predictors of MVC in glaucoma patients. These findings may have significant implications for the identification of drivers with glaucoma at high risk of collisions and also for improving the understanding of driving impairment related to the disease.

## Supporting Information

S1 Table The individual characteristics of each patient included in this study. (XLS)
